# Syphilis from a Sociological Standpoint*Read before the Medical Association of Georgia, April, 1893.

**Published:** 1893-06

**Authors:** Willis F. Westmoreland

**Affiliations:** Atlanta, Ga.; Professor of Surgery Atlanta Medical College


### SYPHILIS FROM A SOCIOLOGICAL STANDPOINT.*

BY WILLIS F. WESTMORELAND, M. D., ATLANTA, GA.

Professor of Surgery Atlanta Medical College.

No matter in what manner you look at this subject it is necessarily of infinite interest, particularly is the origin, one of interest to Americans, as a great many authorities think that the origin of syphilis coincides with the landing of Columbus in Spain, on his return from discovering America. That his sailors contracted the disease in this country, and that it was introduced into Europe by them when they landed at the ports of Lisbon and others (Seville, etc.) in Spain, March 6th, 1493.

If these authors are true, would it not be a terrible blow at the morality and virtue of Europe, if a handful of sailors landing at a few ports in Spain, should inaugurate a disease that in a year or two proved the scourge of Europe. Being declared, as it was, epidemic in 1494, and extending its vicious bar across to France, so that the Parliament of Paris on March 6th, 1497, issued a decree, expelling from Paris all who were suffering from it. Thence across the waters into Great Britain, to such an extent, that the King of Scotland passed a similar decree in the same year.

It seems almost impossible that syphilis could have spread with such rapidity, even acknowledging that it was sometimes much more virulent than at others, allowing for a treatment which at that time probably aggravated rather than cured it, unless a great portion of it was contracted otherwise than by sexual intercourse, and this is the particular point of which I wish to speak.

Naturally in a paper of this scope it is impossible to do otherwise than simply touch upon this subject which has been so vividly impressed upon my mind, by experiences both in clinical and private practice. And how close it comes home to all of us, and how little protection any of us, particularly the laity, have against it!

Year after year, particularly in the clinics, syphilitic subjects,

*Read before the Medical Association of Georgia, April, 1893.

a great many of them women, come before me suffering from this disease in all its stages, from the original lesion to the worst ravages of the second and tertiary stages, with mucous patches and syphilitic ulcers covering them.

And when I find, upon investigation, many of them servants, occupying various household positions as chambermaids, nurses, cooks, waitresses etc., I cannot but think, God protect the family exposed to the baleful contamination of their services, and particularly the poor little defenceless ones whom they nurse, and with whom their relations are so intimate.

Any one who has not investigated this subject, will be overwhelmed with surprise at the number of cases acquired otherwise than by sexual intercourse, and the various means by which it has been transmitted.

Syphilitic poison is readily carried by either the secretions of the initial sore or (chancre), mucous patches, syphilitic ulcerations, or the blood, to an innocent person. Transmitted by direct infection, where any of these secretions of a syphilitic nature come in direct contact with some slight abrasion on the body of a healthy person, or indirect infection, by the secretions adhering to wearing apparel, instruments, or the various articles of household conveniences, and which prove the various means of transmitting the infectious poison to the unsuspecting innocent. The list of these articles is appalling.

Physicians and dentists are themselves exposed, and likewise expose their patients to these dangers. Thousands of physicians and midwives, in the practice of their calling, in examining and operating on syphilitic subjects, or by *post mortem*, are exposed. Dentists are likewise exposed by having their fingers scratched against a rough tooth or instrument, while working on a patient whose mouth is filled with mucous patches.

On the other hand, many more patients are infected by the carelessness of physicians. They may examine patients with an unsuspected chancre on their finger, or with carelessly cleansed hands or instruments, for some of these secretions are thick and tenacious, and when dried cannot be removed by ordinary washing. Think of sixty patients being inoculated with this disease, as the result of eustachian catheterization in the practice of one person. Ten innocent helpless babies, inoculated at one sitting, by being vaccinated from a syphilitic child!

It is a shame to our profession, that the literature of this subject is filled with these cases. It is worse than shameful, for it is the result of criminal carelessness.

And now we turn to another class of cases, where we are also to blame, though not so much as in the former. When patients first come to us with syphilis, they are frequently in the most abject state of horror, as to what the results will be, believing that they are tied down for life by a loathsome disease, for they have viewed every symptom through a magnifying glass.

In trying to relieve their minds of this absolute dejection, the physician is apt to make light of the symptoms and disease. The patients in time, as their mental and physical sufferings are alleviated, do the same, and begin to neglect both treatment and the precautions you have emphasized upon them to take, that they may not transmit this disease to their relations, friends, or associates. Particularly is this true of the lower classes. Others will wilfully disregard all injunctions you may give them.

It is a horrible idea that possibly some relative or dear friend is at this moment employing a syphilitic nurse for his children, who is affectionate, and fond of caressing them, who chews their chewing gum till it is soft, so it won't hurt the poor little one's teeth, who always tries the nursing bottle first to see if baby's milk is of the right temperature, and who is fond of dearie's rubber rings, or a chambermaid who not only touches every article about the house, but possibly uses them, perchance your toothbrush.

Perhaps a cook, who prepares every article of food, washes and handles the dishes, knives and forks, eating out of them, drinking out of the same cups and glasses, and is possibly a little careless about the amount of water she uses in cleaning them. Maybe a young man with a mucous patch on his lips, calling on his sweetheart, who wipes a chapped lip with a handkerchief he has used on his own, or kisses her good night. Instead of his sweetheart, it may be his mother or sister who is the unfortunate sufferer, and presents herself to us later with a chancre on her lip. There are numbers of such cases.

Think of these poor unfortunates "cursed" by the heritage of the sin they have not sinned! I think it justifiable for the physician to warn those most interested, where the patient continues to disregard and ignore the precautions you tell them to take. But we will not discuss this, as this paper is only written to call your attention to the evil and not the remedy.

Sadly we close with a few words on hereditary syphilis. We have nearly all seen these cases. The syphilitic husband, who in spite of our earnest advice, impregnates his wife. And still sadder the man, who regardless of our counsel as to the disastrous results, marries some sweet innocent woman, the one who is all the world to him, (we have all seen them,) afterwards coming to us, sad-faced and hollow-eyed, showing the watchful strain of anticipations they have been under, and they begin to appreciate our words are coming true. We have all listened to them as with trembling lips they tell us, they could not realize, did not dream of this heart-rending occurrence. That they would have died first. Then they feel their error. Now they see what their love was, the

> Love, that breeds its own decay,
> Rotten, ere the blossom burst.

Then we go to the poor unfortunate wife, and do what we can for her, sometimes able to accomplish something, most frequently not. And in a few weeks or months, deliver her of a syphilitic child. And when we return and see the mother hugging and kissing and cooing to the horrible looking infant, for no matter how ugly one begotten, they love and cherish them all the same, for whom there will soon be another laceration of the heartstrings, caused by its loss.

We can but think:

> Blest is the babe that dies within the womb;
> Blest is the corpse which lies within the tomb;
> And, blest that death for which this life makes room.

But even then the sad echoing refrain of anguish wrung from a mother's tender heart is borne to our ears,

> But dreary is the tomb where the corpse lies;
> And wretched is the womb where the child dies;
> And cursed that death which steals this life's disguise.

